# Computational Modelling and Big Data Analysis of Flow and Drug Transport in Microfluidic Systems: A Spheroid-on-a-Chip Study

**DOI:** 10.3389/fbioe.2021.781566

**Published:** 2021-11-23

**Authors:** Sina Kheiri, Eugenia Kumacheva, Edmond W.K. Young

**Affiliations:** ^1^ Department of Mechanical and Industrial Engineering, University of Toronto, Toronto, ON, Canada; ^2^ Department of Chemistry, University of Toronto, Toronto, ON, Canada; ^3^ Institute of Biomedical Engineering, University of Toronto, Toronto, ON, Canada

**Keywords:** organ-on-a-chip, cancer spheroids, drug delivery, full-factorial experiments, microfluidic design space exploration, hierarchical clustering

## Abstract

Microfluidic tumour spheroid-on-a-chip platforms enable control of spheroid size and their microenvironment and offer the capability of high-throughput drug screening, but drug supply to spheroids is a complex process that depends on a combination of mechanical, biochemical, and biophysical factors. To account for these coupled effects, many microfluidic device designs and operating conditions must be considered and optimized in a time- and labour-intensive trial-and-error process. Computational modelling facilitates a systematic exploration of a large design parameter space *via* in silico simulations, but the majority of in silico models apply only a small set of conditions or parametric levels. Novel approaches to computational modelling are needed to explore large parameter spaces and accelerate the optimization of spheroid-on-a-chip and other organ-on-a-chip designs. Here, we report an efficient computational approach for simulating fluid flow and transport of drugs in a high-throughput arrayed cancer spheroid-on-a-chip platform. Our strategy combines four key factors: i) governing physical equations; ii) parametric sweeping; iii) parallel computing; and iv) extensive dataset analysis, thereby enabling a complete “full-factorial” exploration of the design parameter space in combinatorial fashion. The simulations were conducted in a time-efficient manner without requiring massive computational time. As a case study, we simulated >15,000 microfluidic device designs and flow conditions for a representative multicellular spheroids-on-a-chip arrayed device, thus acquiring a single dataset consisting of ∼10 billion datapoints in ∼95 GBs. To validate our computational model, we performed physical experiments in a representative spheroid-on-a-chip device that showed excellent agreement between experimental and simulated data. This study offers a computational strategy to accelerate the optimization of microfluidic device designs and provide insight on the flow and drug transport in spheroid-on-a-chip and other biomicrofluidic platforms.

## Introduction

Over the past few decades, microfluidics (MFs) has emerged as a powerful platform for fundamental and applied research in cell biology, soft robotics, medicine, drug screening, materials science, and analytical chemistry (Whitesides, 2006; Tian and Finehout, 2009; Young et al., 2012; Pak et al., 2015; Rajendra et al., 2015; Moore et al., 2017; Humayun et al., 2018; Khuu et al., 2019; Gevorkian et al., 2021). For all applications, the design of an MF device with optimized geometry is a vital first step of the engineering process, but the optimization process is usually conducted in a trial-and-error manner and is, therefore, time- and labour-intensive (Chakraborty, 2010; Huang, 2013). Many factors have to be considered and tested, such as the geometry and dimensions of microchannels or microwells, operating conditions such as fluid flow rates, and fluid properties. It is highly desirable to utilize a rational approach to the design process that would reduce the number of experimental trials and make it more time-efficient.

Computational modelling enables systematic exploration of a large parameter space *via* in silico simulations aimed at the optimization of the design and operation of an MF device prior to experiments (Enderling and Rejniak, 2013). In silico simulations have been extensively used for lab-on-a-chip platforms to simulate fluid flow and transport phenomena, including droplet formation (Sontti and Atta, 2017, 2020; Mohamed et al., 2019), micro-mixing (Zhang et al., 2008; Suh and Kang, 2010), bacteria growth and culture (Hohne et al., 2009; Westerwalbesloh et al., 2015; Kim et al., 2019; Kheiri et al., 2020), particle sorting and separation (Han et al., 2015; Lu and Xuan, 2015; Amin Arefi et al., 2020; Fallahi et al., 2020), biomechanical forces (Kim et al., 2015; Rousset et al., 2017), and drug delivery (Hossain et al., 2012; Soltani and Chen, 2012; Soltani et al., 2016; d’Esposito et al., 2018). In the specific case of MF devices for cell biology studies, computational fluid dynamics (CFD) was used to investigate molecular transport in a bilayer membrane-based MF device and examine the effect of flow-induced shear stress on endothelial cells (Wong et al., 2017). The CFD model provided guidelines for the design of MF devices operating within a range of physiological shear stresses while ensuring efficient transport of biomolecules through the membrane. In another computational study, a mathematical model coupled with numerical simulations led to an optimal design of MF devices for studies of endothelial cell migration and angiogenesis (Kuzmic et al., 2019). A computational framework was established to elucidate the effect of MF device geometry on cell migration and angiogenesis in a double-channel design with interconnected migration ports. Several studies have focused on the simulations of drug delivery to multicellular tumour spheroids (MCTSs) in a spheroid-on-a-chip platform (Kim et al., 2008; Moshksayan et al., 2018). More specifically, these simulations explored the effect of MF device geometry including microwell and microchannel dimensions on drug supply and uptake by MCTSs. Recently, a numerical study of the design of a tumour-on-a-chip platform was performed to determine its optimal performance in screening multidrug combinations (Hajari et al., 2021).

In the vast majority of numerical studies, the in-silico models are generally applied to a small set of conditions or parametric levels and employed only for “one-factor-at-a-time” (OFAT) experimental designs. For example, in modelling and simulating bacteria growth and glucose transport to bacterial colonies on-chip, the effect of glucose concentration was simulated while all other parameters were maintained invariant (Westerwalbesloh et al., 2015). Similarly, the effect of fluid flow and viscosity was modelled to study cancer cell loading in microwells of the MF device (Hajari et al., 2021). Inlet fluid flow rate and viscosity were varied systematically across three different values and thus nine parameter combinations were used overall, but the geometric characteristics of the MF device and MCTS properties were left constant to reduce the parameter space. Generally, computational models were treated as in silico analogs of experimental setups to simulate an experiment that could have been conducted using standard OFAT or factorial experimental designs. While such numerical studies hold great promise by i) simulating the output result without running an experiment and ii) providing a greater analytical power than is possible with experiments, these models would offer an even greater benefit if a larger parameter space could be explored. By definition, full factorial design (FFD) investigates the effects of all the selected factors and their interactions on the outcome(s) of the experiment (Das and Dewanjee, 2018). The term “full-factorial” refers to the fact that once the various factors of interest and the different levels within each factor have been selected, every conceivable combination of levels in the parameter space will be simulated and analyzed; this is distinct from “fractional factorial design,” where only a fraction or subset of the parameter space is tested and analyzed. Such simulations may reveal the most effective design and operating parameters that have not been considered or experimentally explored. For spheroid-on-a-chip MF platforms, the need for optimization is particularly important, as modifications of the geometry of the MF devices can significantly affect cell culture microenvironment and, subsequently, impact cell aggregation and MCTS structure and fate (Young and Beebe, 2010; Ong et al., 2017; Zuchowska et al., 2017). An approach in which MCTS culture and drug delivery conditions are “screened” in a time- and labour-efficient manner would be highly advantageous, as numerous operating conditions and geometrical constraints create a large parameter space in MF spheroid-on-a-chip platforms. Here, we report an efficient computational strategy for simulating fluid flow and transport of low-molecular-weight solutes (drugs) in an MF spheroid-on-a-chip platform (Wang et al., 2016). The platform is comprised of a large array of MCTSs compartmentalized in cylinder-shaped microwells connected with a common channel used for the continuous supply of cell culture medium and/or drug solution to the MCTSs (Ota et al., 2011; Schneider et al., 2013; Wang et al., 2016). We show that based on (>15,000) MF device designs and flow conditions acquired in a single dataset across a large parameter space (operating conditions, geometrical constraints, and different material porosity), these simulations can be effectively utilized to identify the most effective MF device design. The computational strategy combined and utilized four key factors: i) the governing physical equations; ii) parametric sweeping; iii) parallel computing; and iv) extensive dataset analysis, which all together enable a complete “full-factorial” exploration of the design parameter space in a combinatorial fashion. Importantly, the simulations were conducted in a time-efficient manner without requiring massive computational time. The computational model integrated multiple physical interfaces to examine advective and diffusive modes of drug delivery to MCTSs in MF devices with various geometries and examined the impact of MF device design on drug transport. This study demonstrates the power of the computational strategy and offers insights on drug transport phenomena in MF devices under a broad range of experimental conditions and MF device designs.

## Materials and Methods

**FIGURE 1:**
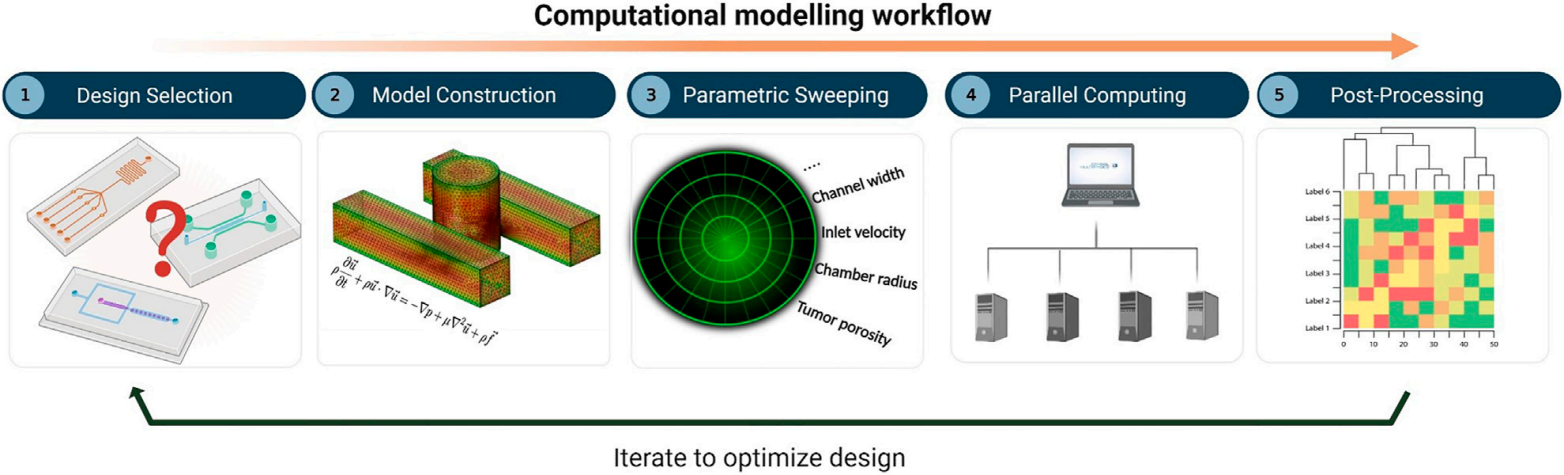
Computational modelling workflow for microfluidic design optimization, involving: (1) design selection, (2) model construction including mesh generation, specification of governing equations, and selection of parametric levels, (3) parametric sweeping, (4) parallel computing, and (5) post-processing and analysis.

The overall workflow of the computational framework (Figure 1) consists of five major stages: i) definition of the MF device geometry or design selection, ii) construction of the in-silico model, which involves mesh generation, specification of the governing equations, and selection of the values or levels of all factors, iii) parametric sweeping across the entire parameter space, iv) parallel computing, and v) post-processing and analysis. The computational model was built using COMSOL Multiphysics 5.5 software (COMSOL, Stockholm, Sweden), a finite-element-based commercial CFD package. To capture the set of governing equations needed to model flow and molecular transport, two distinct COMSOL physics interfaces were coupled together, namely 1) the Laminar Flow (*spf*) interface and 2) the Transport of Diluted Species (*tds*) interface. An automatic solution-adaptive mesh refinement tool was used to regenerate the mesh and maintain mesh quality within an acceptable range for all simulations (Supplementary Material). The criterion for convergence in the simulations was selected to be a drop of four orders of magnitude $\left(10^{-4}\right)$ .

### MF Device Geometry: Our Spheroid-on-a-Chip Model

**FIGURE 2:**
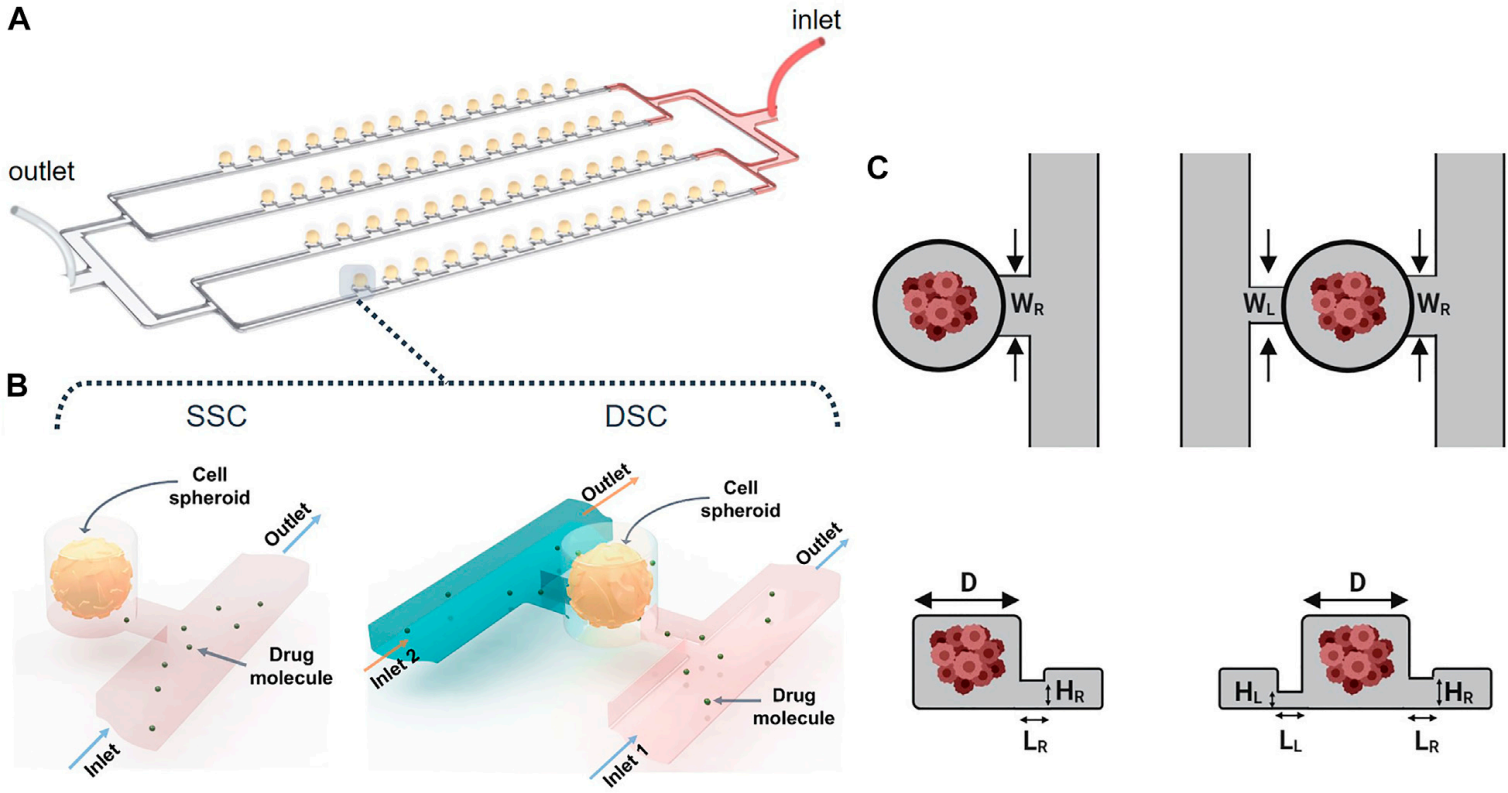
Spheroid-on-a-chip device design and geometry. **(A)** 3D illustration of the array-based spheroid-on-chip microfluidic device used in this study. **(B)** Illustration of single supply channel (SSC) and double supply channel (DSC) designs of the spheroid-on-a-chip device. **(C,D)** Geometric parameters considered in the design optimization of the SSC and DSC spheroid-on-a-chip designs.

Array-type MF devices have been extensively used for spheroid formation and drug screening (Liu et al., 2015; Sabhachandani et al., 2016; Moshksayan et al., 2018). They can accommodate hundreds of cell-laden droplets in the microwells (Wang et al., 2016; Huang et al., 2017). As a case study, our computational study focused on a particular design shown schematically in Figure 2. The MF device consists of parallel rows of cylindrical microwells positioned on a side of a microchannel “supply line.” The parallel rows of channels are connected to a single inlet and a single outlet *via* serial divisions. Cell-laden droplets are compartmentalized in the microwells, and subsequently, transform into MCTSs embedded in a hydrogel scaffold. The geometry of the microwells determines droplet size, which in turn, controls MCTS dimensions (Kaminski and Garstecki, 2017). This MF platform has the following advantages: i) the capability to form uniformly sized MCTSs, ii) the ability to monitor individual MCTSs in real-time, and iii) long-term MCTS culture, enabled by the use of a suitable hydrogel scaffold. Because droplet generation is controlled by the relationship between the channel geometry and the microwell aspect ratio (Schneider et al., 2013), the design of such MF devices to study, for example, the biomechanical forces exerted on the MCTSs or drug delivery to the MCTSs is a challenging task. While the effect of MF device geometry on droplet formation has been studied (Cohen et al., 2010), the effect of these constraints on fluid flow and transport of biological molecules such as drugs or nutrients has yet to be examined (Lee et al., 2012; Schneider et al., 2013; Wang et al., 2016).

Here, our goal was to simulate the effects of geometrical and non-geometrical constraints on the transport of drugs and nutrients to the arrays of MCTSs in the spheroid-on-a-chip MF platform illustrated in Figure 2A. In all simulations, MCTS diameter was 100 µm. To demonstrate the versatility of our computational strategy, we studied both the single-supply-channel (SSC) design (shown in Figure 2A) and a modified double-supply-channel (DSC) design that has not been reported (Figure 2B). Five different geometric parameters of the MF device were examined, each with a specific range of values (Table 1 **)**.

**TABLE 1:** Geometrical, material, and operating conditions used in current spheroid-on-a-chip design study.

Parameter	Notation	Range	Steps	Number of conditions	References
Inlet velocity left	Q_1_	0.01–0.02 ml/h	0.01, 0.02 ml/h	2	Patra et al. (2016); Moshksayan et al. (2018)
Inlet velocity right	Q_2_	0.01–0.02 ml/h	0.01, 0.02 ml/h	2	Patra et al. (2016); Moshksayan et al. (2018)
Infusion width left	W_L_	∼0–60 µm	0.1, 20, 30, 40, 60 µm	5	Cohen et al. (2010); Ota et al. (2010); McMillan et al. (2016)
Infusion width right	W_R_	20–60 µm	20, 30, 40, 60 µm	4	Cohen et al. (2010); Ota et al. (2010); McMillan et al. (2016)
Infusion height left	H_L_	∼0–60 µm	0.1, 20, 30, 40, 60 µm	5	Cohen et al. (2010)
Infusion height right	H_R_	20–60 µm	20, 30, 40, 60 µm	4	Cohen et al. (2010)
Chamber radius	R	80–160 µm	80, 120, 160 µm	3	Cohen et al. (2010)
Porosity	φ	0.2–0.9	0.2, 0.5, 0.7, 0.9	4	Hwang et al. (2010)

### Governing Physical Equations

Molecular transport was modeled as time-dependent and three-dimensional, and included advection and mass transfer in the porous domain. Molecular transport was thus governed by the advection-diffusion equation:

$$\frac{\partial c}{\partial t}=S+D\nabla^{2}c-u\cdot \nabla c$$ (1)

where  c is the species concentration (mol/m^3^),  S is the supply influx (mol/m^3^⋅s),  u is the fluid velocity (m/s), and D is the diffusion coefficient of the molecular species (m^2^/s). Fluid flow was considered time-dependent, three-dimensional, laminar, and incompressible, and was thus governed by the continuity and Navier-Stokes equations:

$$\frac{\partial \rho }{\partial t}+\nabla .\left(\rho u\right)=0$$ (2)

$$\rho \left[\frac{\partial u}{\partial t}+u\cdot \nabla u\right]=-\nabla p+\mu \nabla^{2}u+\rho f$$ (3)

where  ρ is the fluid density (kg/m^3^); u is the fluid velocity (m/s),  p is the pressure (Pa), μ is the dynamic viscosity of the fluid (Pa⋅s), and f is the body force per unit volume (N/m^3^). Eqs 2 and 3 represent conservation of mass (continuity equation) and momentum (Navier-Stokes equations), respectively. Here we assumed the body force term was zero as we ignored gravity and assumed there were no other external field-induced forces present. To study drug transport and penetration into spheroids, the spheroids in our simulations were treated as porous media (defined as a “matrix domain” in COMSOL) surrounded by the primary fluid domain where MF flow was occurring. Porous media flow within the spheroid was governed by:

$$\frac{\partial }{\partial t}\left(\rho \varepsilon_{p}\right)+\nabla .\left(\rho v\right)=Q_{m}$$ (4)

where $\varepsilon_{p}$ is the porosity (or void fraction) of the matrix domain,  v is the velocity inside the matrix domain, and $Q_{m}$ is a mass source within the matrix domain (kg/m^3^·s). The velocity field within the porous domain can be calculated using Darcy’s law:

$$v=-\frac{\kappa }{\mu }\nabla p$$ (5)

where κ and μ are the permeability of the porous material (m^2^) and dynamic viscosity of the fluid material (Pa⋅s), respectively. Solving the velocity field within the matrix domain determined the advection mode of drug transport. The diffusion mode of transport, in contrast, was calculated using Fick’s law:

J=−D∇c (6)

where  J is the diffusive flux (mol/m^2^⋅s), which points in the negative direction of the concentration gradient. Finally, drug transport was solved through mass conservation in which both advection and diffusion modes of transport were included using the following:

$$\frac{\partial c}{\partial t}+\nabla \cdot J+v\cdot \nabla c=R$$ (7)

where R is the drug reaction rate (mol/m^3^⋅s). This reaction rate was set to zero in all our simulations.

### Parametric Sweeping

To tackle this large combinatorial problem of >15,000 modelling scenarios, we employed parallel computing using the CAD Compute Cluster from CMC Microsystems and divided the various scenarios into smaller subsets to be run simultaneously on different parallel processors (Afzal et al., 2017). Using this large computational resource (eight nodes, Dual 14-core, 2.4–3.3 GHz CPU), the total computational time was reduced ∼10-fold compared to running the same CFD simulation on a single desktop workstation (7-core, 2.1–3.2 GHz CPU).

### Post-Processing and Analysis

For all simulations, pressure, velocity, and concentrations were analyzed and evaluated in 2D and 3D configurations. The 2D and 3D plots were generated using COMSOL Multiphysics plotting functions. For 1D plotting and investigation, the data were extracted and exported from the CFD model using volume averaged evaluation functions. Five key metrics of flow and transport in the spheroid domain were extracted: concentration, velocity, shear rate, advective flux, and diffusive flux. These metrics were normalized between 0 and 1 before being compiled into heatmaps. Clustered heatmaps were generated based on the exported data and post-processing by the Seaborn library and clustering functions in Python (Supplementary Material). For convenience of describing specific device designs, we established a naming convention based on the concatenation of the six design parameters selected for the given design. An executable standalone application was also designed and created using the application builder module in COMSOL Compiler (Supplementary Material).

## Results and Discussion

### Velocity and Pressure Distribution in a Single Row of Microwells

We conducted numerical simulations with our model to examine the effects of different parameters on biomechanical forces, fluid transport, and drug uptake in and around tumour spheroids cultured on-chip. First, a single row of the device, which includes 50 microwells, was modeled to determine the steady state pressure gradient and velocity field in the microchannel (Supplementary Figure S4). Using a prescribed inlet flow rate of 0.01 ml/h, we found the maximum pressure in the microchannel to be 17.5 Pa and confirmed that the pressure drop between any two adjacent microwells was consistent along the entire length of the microchannel (Supplementary Figure S4A). Fluid velocity inside the microwells was ∼10× lower compared to the fluid velocity in the main microchannel (Supplementary Figure S4B **)**. Notably, the velocity magnitudes inside the chambers were found to be ∼0.02 mm/s (Supplementary Figure S4C), consistent with typical physiological conditions in tumour tissue (Chary and Jain, 1989).

**FIGURE 3:**
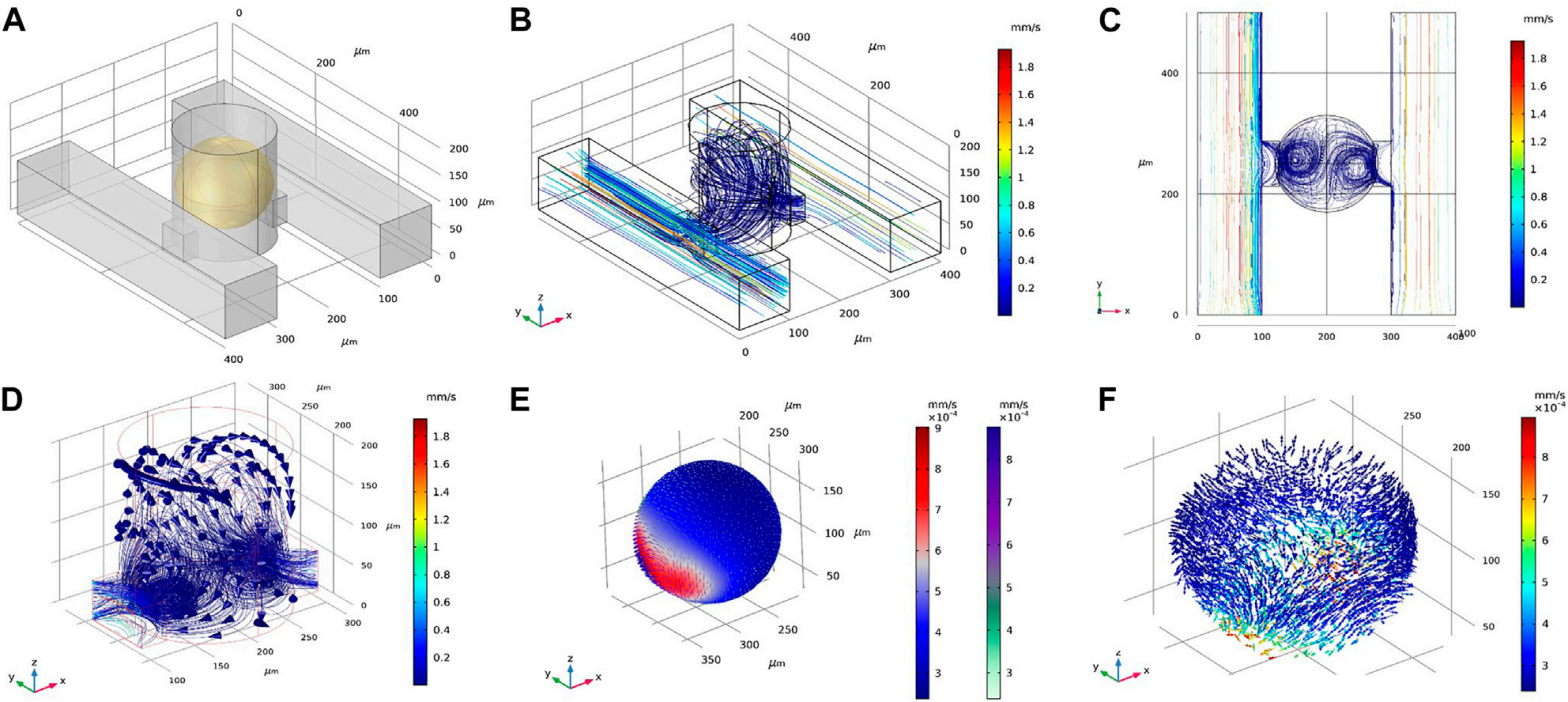
Representative results of flow and transport simulations in COMSOL. **(A)** A single unit of the double-supply channel (DSC) design of the spheroid-on-a-chip device, consisting of the microwell for spheroid formation and culture, two supply channels, two connecting channels, and the spheroid itself (shown in yellow). **(B,C)** Velocity streamlines in the DSC device. **(D)** Velocity vectors showing fluid flow circulation overlaying streamlines of flow in the microwell. **(E)** Velocity magnitude of fluid flow on the surface of the spheroid, shown as arrow surface and surface contours. **(F)** Velocity vectors within the spheroid (treated as porous medium).

To explore a larger design space, we examined the DSC design consisting of two parallel supply microchannels on either side of the spheroid-containing microwells (Figure 3A). Note the DSC design consisted of the same set of geometrical parameters and range of values as the SSC design. Flow rates for both channels were first prescribed at 0.01 ml/h. Under this condition, velocity magnitudes inside the spheroid chambers were ∼0.02 mm/s, similar to the SSC design (Figure 3B). We examined fluid flow circulation inside the microwell (Figures 3C,D **)** and found that the streamlines formed two flow vortices that surrounded the spheroid. These vortices in the DSC device were not observed in the SSC design **(** Supplementary Figure S2 **)**. Furthermore, adding the second supply microchannel not only improved circulation of the fluid inside the microwell, but also improved the uniform distribution of surface shear forces on the spheroid (Figure 3E) compared to the SSC design (Supplementary Figure S2). We next simulated fluid flow inside the spheroids (treated as a porous medium) to better understand the effects of adding the second supply microchannel to flow through the internal spaces of the spheroid (Figure 3F). We found that fluid motion had two high velocity regions corresponding to the locations closest to the supply microchannels on the two sides, while the rest of the spheroid observed velocity magnitudes that were an order of magnitude lower. Taken together, these initial simulations revealed several major differences between SSC and DSC designs that motivated further exploration into ways to optimize the design to achieve maximum drug transport efficiency for the purposes of developing a rapid high-throughput spheroid drug screening tool. Before conducting such a massive design space exploration, however, we experimentally validated our computational model by using the actual spheroid-on-a-chip platform and the select experimental flow conditions.

### Experimental Validation of the Computational Model

**FIGURE 4:**
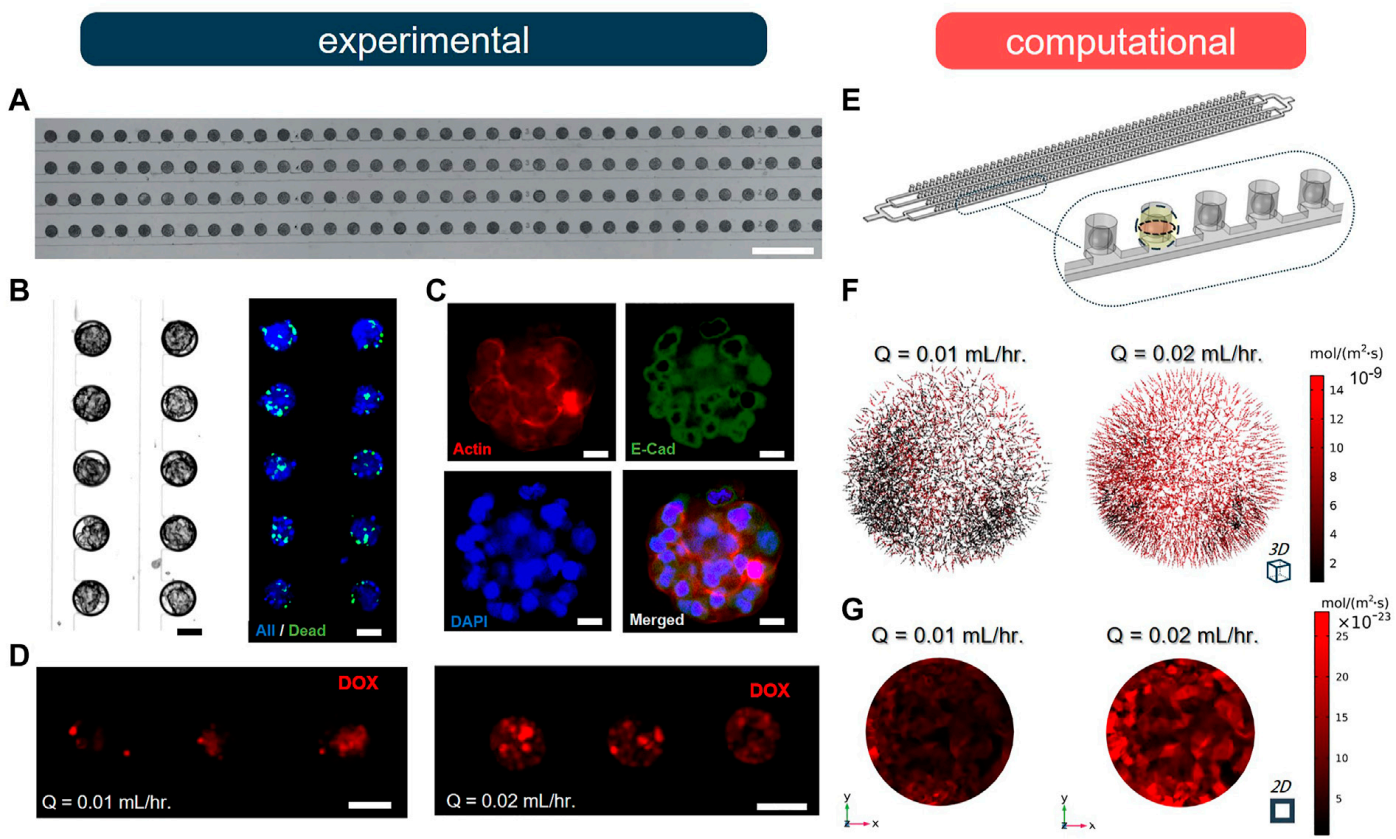
Doxorubicin (DOX) uptake by spheroid under two different flow rates (*n =* 5). **(A)** A fragment of the 100 μm-diameter arrays of MCF-7 breast cancer spheroids after loading. Scale bar is 1 mm. **(B)** Brightfield and fluorescence (NucGreen^®^/NucBlue) images of formed MCF-7 breast cancer spheroids after 72 h cell culture. Scale bar is 50 µm. **(C)** Immunofluorescence staining of tumour spheroids after 72 h of cell culture by E-cadherin antibody (green), DAPI (blue) and F-actin (red). Scale bar is 25 µm. **(D)** Fluorescence images of spheroids after 2 h perfusion of 10 µM DOX under different flow conditions. Scale bar is 100 µm. **(E)** The defined geometry and porous medium domain in COMSOL Multiphysics. **(F)** Results of drug penetration within the spheroid are visualized as arrows based on the total flux of molecules. **(G)** Results of total flux of DOX evaluated in the midplane of spheroid when Q = 0.01 ml/h and 0.02 ml/h.

We conducted a series of experiments to validate our computational model, using an SSC array-type MF device to form MCF-7 breast cancer MCTSs and examine the effect of different flow rates on drug uptake. The experimental MF device contained 200 MCTSs in the microwells that were organized in four parallel rows (Figure 4A). The microwells were connected to a common channel used to supply cell culture medium and drug treatment [for more details on fabrication, MCTS formation, and cell staining protocols see Supplementary Material and (Chen et al., 2021; Prince et al., 2021)]. The cell suspension occupied 100% of the microwells in the form of cell-laden droplets; MCTSs formed within 72 h of cell culture. The viability of breast cancer cells in the MCTSs was evaluated using NucBlue (Hoechst 33342, nuclei) and NucGreen (dead cells), showing ∼89% viability throughout the entire spheroid array (Figure 4B **)**. We also confirmed the expression of E-cadherin and F-actin *via* immunostaining to examine proper localization of cell-cell junctions and the presence of actin cytoskeleton, respectively (Figure 4C). Next, MCTSs were treated with 10-µM Doxorubicin (Dox) under two different flow rates, Q, of 0.01 and 0.02 ml/h. The results showed that after 2 h of drug treatment by perfusion, drug uptake at Q = 0.02 ml/h was significantly higher than the uptake at Q = 0.01 ml/h (Figure 4D). To validate our computational model, we simulated the experimental geometry and flow conditions in silico using the same corresponding geometry and conditions **(** Figure 4E). As predicted, the fluxes of drug molecules into the MCTSs at Q = 0.02 ml/h were significantly higher than at Q = 0.01 ml/h (Figure 4F **)**. The increase in flow rate resulted in approximately five-fold increase in the total flux of the drug. Moreover, we compared and analyzed the penetration of drug molecules into the MCTSs using the CFD model (Supplementary Material). The results revealed that the increase in the flow rate of drug treatment significantly enhanced the penetration of the MCTSs with a drug (Figure 4G **)**. Thus, the experiments validated our model, thereby enabling us to study changes in drug transport under different conditions and uncover optimal MF device design parameters for a particular application.

### Using the Computational Model as a Design Tool

To better understand and compare the effects of device design on drug delivery and biomechanical forces on the spheroids, we used a systematic computational approach that allowed the exploration of >15,000 device designs and flow scenarios in a single simulation dataset. Our computational model covered a large parameter space representing a full-factorial experimental design, the results of which can be used during the design stage of the MF device to study the impact of design changes on drug transport. In total our complete dataset includes 10 billion datapoints (∼15,000 scenarios × $\left(∼7\times 10^{5}\right)$ finite elements per fluid flow and porous medium domain) resulting in ∼95 GBs of data. In the following sections, we describe results from the massive dataset, where we simulated the drug transport of Dox, a widely used drug for tumor therapy (Mehta et al., 2012), as our model drug.

### Drug Permeation Depends on Spheroid Porosity

**FIGURE 5:**
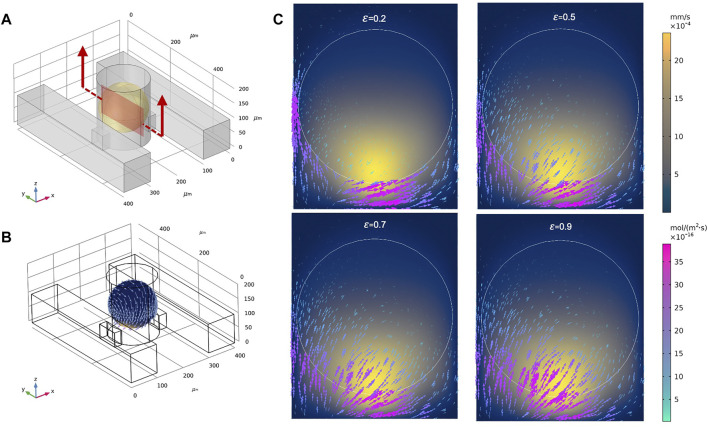
Total molecular flux and velocity magnitudes for different spheroid porosities. **(A)** A single unit of the double-supply channel (DSC) design of the spheroid-on-a-chip device, where the defined cross-section represents the sagittal yz-plane. **(B)** 3D contours of velocity magnitude and 3D vectors of drug flux around the spheroid when φ = 0.9. **(C)** Velocity magnitudes (2D surface plot) and vectors of drug flux within the sagittal yz-plane for φ = 0.2, 0.5, 0.7, and 0.9.

Our parametric “design screen” of >15,000 simulations enabled us to systematically investigate the effects of individual parameters on drug uptake *a posteriori*. One physical property of spheroids that plays an important role in drug delivery is the spheroid porosity (Soltani and Chen, 2012; Soltani et al., 2016). Here, we focused on the effects of spheroid porosity on the velocity field and drug delivery inside the spheroid when the inlet flow rates Q_1_ and Q_2_ were both 0.01 ml/h, and considered advective and diffusive modes of transport separately. We studied the effects of porosity by extracting the simulated results in DSC designs and four different porosities, ε=0.2 , 0.5, 0.7, and 0.9, while keeping the geometry, fluid flow, and drug properties the same. We found that changes in porosity increased the total flux of the drug by ∼17× when porosity increased from ε=0.2 , to ε=0.9 (Figure 5C). However, velocity magnitudes (as viewed on the sagittal *yz* plane) were similar across all porosities. Thus, spheroid porosity clearly influences the drug/nutrient delivery, but has minimal effect on the velocity magnitudes within the spheroid.

### Drug Permeation Depends on Mode of Transport

Diffusive and advective fluxes are concurrent processes in biological tissues where fluid flow exists, such as the interstitial flow within the stroma. Studying both diffusion and advection transport modes in the spheroid-on-a-chip provides insight into the dynamics of drug uptake while also opening the door to applying our computational models for novel drug design based on transport efficiency (Minchinton and Tannock, 2006; Dewhirst and Secomb, 2017). The delivery of adequate concentrations of anticancer drugs to cancer site strongly depends on the structure and drug transport mechanism in tissue. While the effects of structure on tumor response has been studied (Steuperaert et al., 2017; Li et al., 2021), the effect of transport-related factors that play an important role in the delivery of anticancer drugs are much less studied (Dewhirst and Secomb, 2017). Our model considered both diffusion and advection modes of transport separately, facilitating the evaluation of drug transport mechanisms in our different device designs.

**FIGURE 6:**
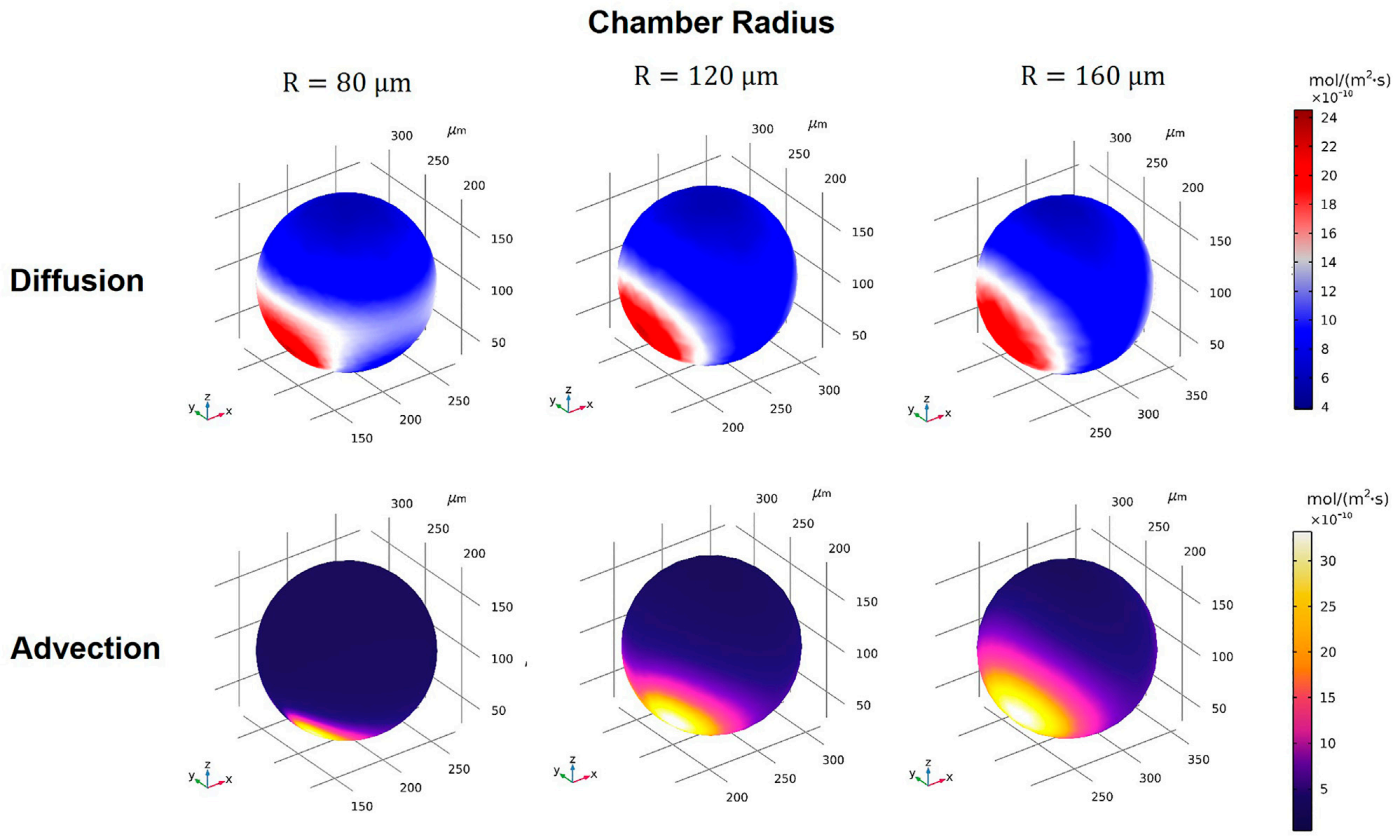
3D volume contours of diffusion and advection fluxes for different microwell sizes of R = 80, 120, and 160 μm (W_R_ = H_R_ = W_L_ = H_L_ = 25 µm).

Using our model, we assessed the drug transport modes for Dox under various conditions (see summary of conditions in Supplementary Table S1). First, we arbitrarily selected one device design (W_R_ = H_R_ = W_L_ = H_L_ = 25 μm, Q_1_ = Q_2_ = 0.01 ml/h, ε=0.5 , C_dox_ = 10 µM) and varied only the chamber radius from 80, 120, and 160 µm (the complete dataset is summarized in the sections below). When we compared the advection and diffusion transport modes by visualizing 3D volume contours of flux, we found significant differences between the rates of change in diffusion and advection fluxes (Figure 6 **)**. Closer inspection of the graphs revealed that the transport of drug molecules was diffusion-dominant for smaller microwells (80 μm) but advection-dominant for larger microwells (160 μm). Note that we refer to a transport mode as “dominant” if the volume-averaged flux of the dominant mode is (or exceeds) 10x greater than the volume-averaged flux of the other mode. If the flux ratio between modes is less than 10, we consider it a mixed-mode scenario. Our simulations showed that even a single change in one geometric feature (e.g., chamber radius) can substantially affect the drug uptake mechanisms. These results can be further used as a convenient tool to assist pharmacodynamics studies and help identify limiting factors for drug delivery to spheroids cultured on-chip.

### Drug Delivery Efficiency and Biomechanical Forces

As stated above, adding a second supply microchannel to the device creates vortices inside the microwell and significantly affects the fluid flow around the spheroid. To examine the impact of device geometry and flow patterns on drug transport, we studied drug penetration and uptake by measuring the drug concentration at the center of the spheroid over time using a 3D point probe in COMSOL.

**FIGURE 7:**
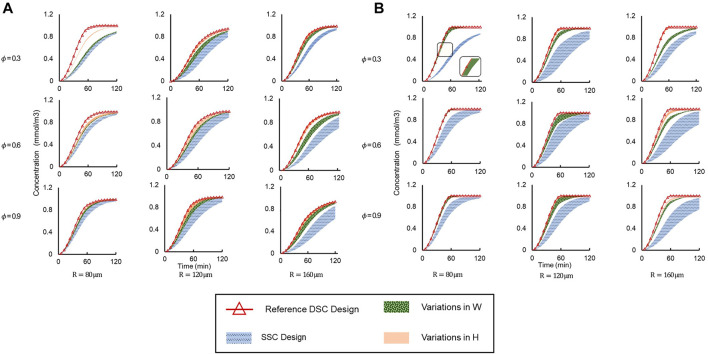
Concentration vs time profiles simulated at the spheroid core up to *t* = 120 min under different geometric conditions and spheroid porosities (*φ*) for **(A)** Q_1_ = Q_2_ and **(B)** Q_1_ = 2 × Q_2_. Legend: “SSC Design”—entire range of SSC designs; “Reference DSC Design”—H_R_ = H_L_ = W_R_ = W_L_ = 60 μm; “Variations in H”—W_R_ = W_L_ = 60 μm are constant and H varies independently; “Variations in W”—H_R_ = H_L_ = 60 μm are constant and W varies independently.

Drug concentration time profiles at the center of the spheroid were plotted for a myriad of different conditions (Figure 7). We applied two different flow conditions of interest, corresponding to when supply channel flow rates were i) equal (Q_1_ = Q_2_) (Figure 7A), and ii) unequal (Q_1_ = 2 × Q_2_) (Figure 7B). For each of these flow conditions, the dataset was further divided by varying spheroid porosity ε (rows) and chamber radius *R* (columns). Finally, for each specific ε and *R*, we plotted four different groups of curves that allowed us to compare effects of geometry: i) all SSC designs, ii) our “reference” DSC design where H_R_ = H_L_ = W_R_ = W_L_ = 60 μm (maximum connecting channel size), iii) varying H_R_ and H_L_ independently (W_R_ = W_L_ = 60 μm), iv) varying W_R_ and W_L_ independently (H_R_ = H_L_ = 60 μm). The “reference” DSC design was chosen arbitrarily from the set of simulated designs to allow convenient comparison between different designs.

For equal supply channel flow rates (Figure 7A **)**, drug delivery was clearly more efficient in all DSC designs compared to the SSC designs, as the drug concentration at the spheroid core was higher for all DSC designs compared to SSC designs in the same amount of time. The reference DSC design was the most efficient, as the spheroid core reached maximum concentration in the shortest amount of time due to the maximum cross-sectional area of the connecting microchannels. Additionally, changing the height of the connector (H_R_ and H_L_) showed a larger effect on drug delivery efficiency compared to changes in the width of the connecter (W_R_ and W_L_). This effect was more apparent in the simulation with a smaller microwell diameter and less porous spheroids. This can be attributed to the fact that by increasing the size of the microwell, the dominant drug transport mode changes from diffusion to advection (as mentioned above), while by increasing the connector height, more drugs will enter the spheroid at a higher location that is closer to the spheroid core.

To explore the effect of flow rate on drug delivery, the same device designs were simulated under the same conditions, but with one supply channel flow rate at twice the flow rate of the other (Q_1_ = 2 × Q_2_). The same trends of drug delivery efficiency were observed (Figure 7B **)**. Interestingly, we observed that unequal flow rates in the supply microchannels further improved drug delivery efficiency compared to equal flow rates, as the amount of drug reaching the spheroid core was higher in the same amount of time for unequal flow rates (Q_1_ = 2 × Q_2_) than for equal flow rates (Q_1_ = Q_2_). For example, when φ=0.6 and *R* = 120 μm, drug concentration at the spheroid core reached a plateau at $1\times 10^{-3}\text{mol}/{\text{m}}^{3}$ at t = 60 min when Q_1_ = 2 × Q_2_, but drug concentration did not reach this plateau for Q_1_ = Q_2_, even after *t* = 120 min. This difference was likely caused by a pressure gradient between two connectors that was generated by the unequal flow rates, ultimately leading to more drugs being actively driven inside the microwell (Supplementary Material).

### Post-Processing and Analysis: Using Design Heatmaps and GUIs as Visualization Tools

**FIGURE 8:**
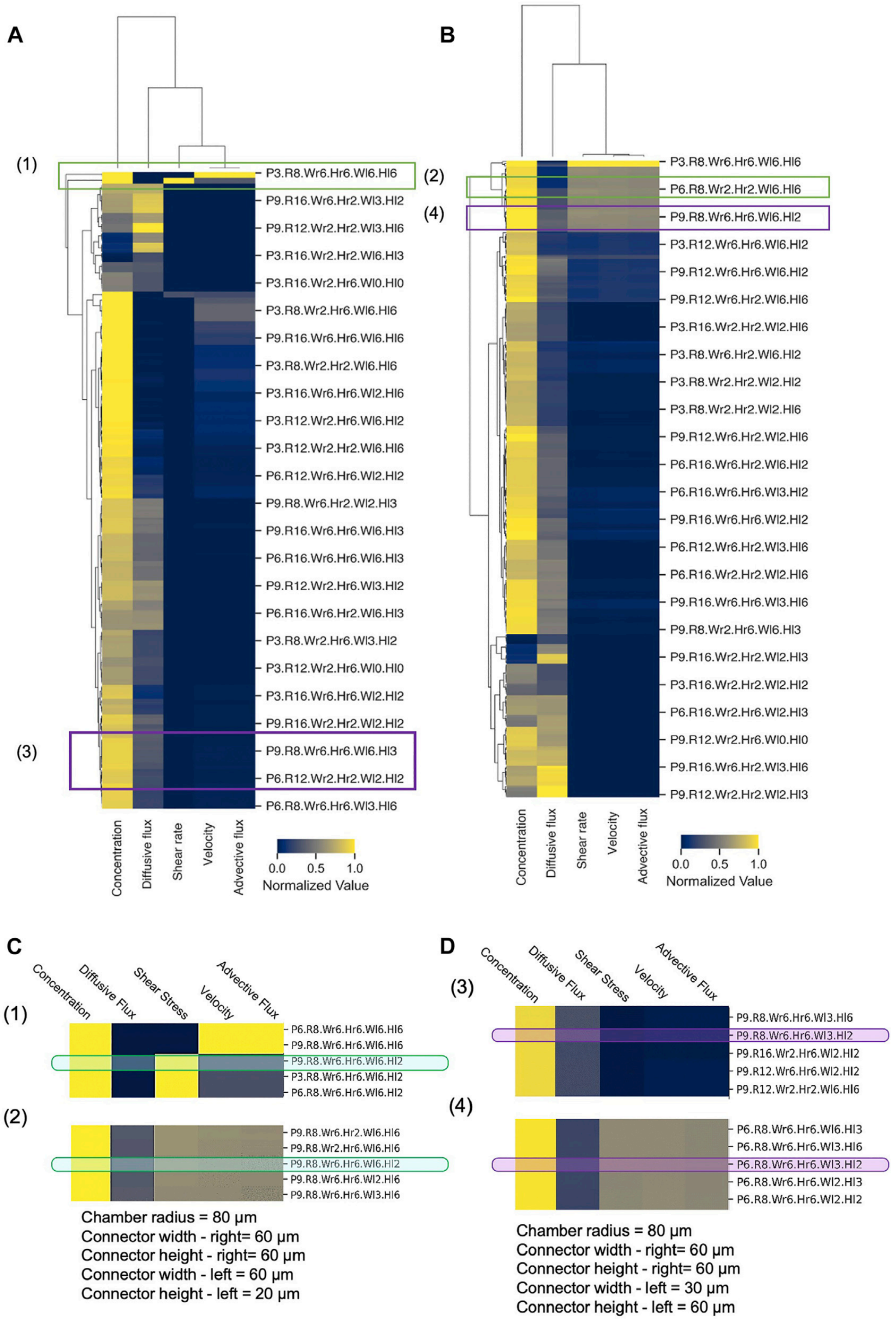
Hierarchical clustering analysis of MF design space. Hierarchically clustered “design heatmaps” for **(A)** Q_1_ = 2×Q_2_ and **(B)** Q_1_ = Q_2_, show normalized values of concentration, shear rate, velocity, and advective and diffusive fluxes as five key metrics (columns). Independent device designs (rows) are designated by a naming convention, where P = Porosity, R = Chamber radius, W_r_ = Connector width—right, H_r_ = Connector height—right, W_l_ = Connector width–left, H_l_ = Connector height—left (e.g., P3. Wr6. R8.Hr6. Wl6. Hl6 denotes *p* = 0.3, W_r_ = 60 μm, R = 80 μm, H_r_ = 60 μm, W_L_ = 60 μm, H_L_ = 60 µm). Hierarchical clusters were computed with Euclidean distance and farthest point algorithm in Python 3.0 (Supplementary Material). **(C,D)** Illustrative examples of device design selection. Green boxes–specific design that achieves both low and high shear by changing flow rate ratio only, with all other metrics constant; Purple boxes–specific design that achieves both advection-dominant and diffusion-dominant drug penetration by changing flow rate ratio only, with all other metrics constant.

Because of the massive dataset generated by our computational design screen, design space exploration became complicated, cumbersome, and time-consuming. To reduce the complexity of the design space and summarize the CFD simulation results in a more convenient fashion, we employed novel classification and clustering methods to sort the various conditions and flow scenarios into groups and reveal underlying patterns in the design parameters **(** Figure 8). To evaluate a given device design, we examined the volume-averaged value of five key metrics of flow and transport in the spheroid domain: concentration, velocity, shear rate, advective flux, and diffusive flux. Hierarchically clustered heatmaps were generated for the cases of unequal supply flow rates (Q_1_ = 2 × Q_2_) (Figure 8A **)** and equal supply flow rates (Q_1_ = Q_2_) (Figure 8B **)**. These heatmaps summarized the five-key metrics for all 15,000 simulations, enabling us to rapidly and succinctly compare different design effects on the transport of drugs in our principal spheroid-on-a-chip layout.

Hierarchical cluster analysis revealed that for both Q_1_ = 2 × Q_2_ and Q_1_ = Q_2_, shear rate, velocity, and advective flux were grouped together as flow and transport metrics, while both concentration and diffusive flux were differentiated from this primary cluster with only moderate similarities between them. As expected, this clustering predicted that modifications of the device design will affect fluid velocity, shear stress, and advective flux with the same tendency, and that these metrics cannot be varied or controlled separately. On the other hand, the heatmap appeared to show that specific designs can have high concentration and low diffusive flux or vice versa, which illustrates the potential to tune the design based on these two metrics. In terms of clustering of the designs, the heatmap showed that generally, in SSC devices, the drug uptake is mainly diffusion-dominant, and the exerted shear stress and velocity on the spheroids are lower compared to the DSC device design. However, the total amount of drug transport in DSC devices appeared to be higher compared to the SSC device, which is in line with the results in Figure 7.

Our hierarchically clustered heatmaps improve our understanding of the effects of different parameters on the metrics of flow and transport in the spheroid-on-a-chip layout and facilitate the selection of design parameters prior to any fabrication or experimentation. When designing MF devices, choosing the right range of parameters requires substantial effort and time. Hierarchical clustering helps narrow the design parameter space and empowers the researcher to quickly find the optimal design solution for a given application. To illustrate, we considered a hypothetical experiment that involved studying the effects of shear stress on drug delivery, and asked whether we could select one specific device design from the heatmap that could a) achieve rapid drug penetration (i.e., high drug concentration in the spheroid core in the shortest amount of time), b) permit both high and low applied shear stress on spheroids by changing operating conditions only, and c) keep all other key metrics relatively invariant to create a controlled experiment. Indeed, when we examined the heatmaps in Figures 8A,B closely, we discovered that the design (P9.R8.W_r_6.H_r_6.W_l_6.H_l_2) satisfied all these criteria (Figure 8C), since high shear stress can be achieved with Q_1_ = 2Q_2_ (Figure 8A) while low shear stress can be achieved with Q_1_ = Q_2_, all in the same geometry. Similarly, as another example, we considered a hypothetical experiment where we examined the effects of advective-dominant vs mixed advective-diffusion transport modes on drug penetration into spheroids. We were able to identify that the design (P9.R8.W_r_.H_r_6.W_l_3.H_l_2) could achieve advective-dominant transport when Q_1_ = Q_2,_ where advection is ∼23× greater compared to diffusive flux. To switch to the mixed mode of transport where advective and diffusion fluxes contribute similarly (i.e., diffusive flux is only 2.3× greater than the advective flux), the same design can be used with Q_1_ = 2 × Q_2_ (Figure 8D). Hence, generating hierarchically clustered heatmaps enabled us to summarize and predict drug delivery distribution and efficiency and identify a range of parameters for a particular set of experiments or applications. Thus, our big data “design heatmap” can ultimately offer a rational approach to the design process that helps us select appropriate designs for proposed experiments in a more time-efficient manner.

Since the primary objective of our computational modelling framework (including our post-processing methods) is to provide the MF engineer with a useful design tool to reduce the design iteration time, guide experiments, and offer operating conditions for end-users of the spheroid-on-a-chip devices, we ventured to create a standalone user-friendly application that can be installed and executed on different devices and operating systems, including Windows, macOS, and Linux (Supplementary Material). This standalone app, which we call “SINAI” (Spheroid-chip Investigation by Numerical modelling-Application Interface), enables engineers, biologists, and other MF researchers to modify and run the simulations directly—even without the need for parallel computing resources—and with full control over geometrical, biomaterial, and fluid parameters. The app allows the sharing and dissemination of computational modelling knowledge widely and openly, including to those who do not have a COMSOL Multiphysics license. We envision that this full-factorial computational framework will have the potential to accelerate MF device design for future spheroid-on-a-chip systems, and more broadly for other organ-on-a-chip systems.

We note that every computational model is by its nature a simplification of the entire system; however, in comparison to experiments, our model covered a comprehensive range of flow rates of liquids, microwell and supplying channel dimensions, and MCTS porosity to provide a broad spectrum of possible combinations that may result in desired or undesired effects for MCTS formation and drug delivery. Typically, the process of parametrical sweeping and its range is limited to the computational techniques and capacity. In our *in-silico* model, we utilized parallel computing on a Cloud cluster to improve the performance of this sizeable computational task in a reasonable time. Sweeping and post-processing over the parameter space must be performed for each independent design, which will lead to thousands of design modifications that represent refinements of the principal design. It is conceivable to repeat the process for as many different conceptual designs as needed for the given design problem. Indeed, our approach cannot generate completely novel design concepts automatically: the microfluidics design engineer must continue to play the creative role of generating innovative design concepts that can then be refined extensively using our modelling workflow. In the case described here, we conceptualized SSC and DSC configurations first, and then utilized our modelling and post-processing approach to determine optimized design and operating parameters.

Our work focused on the interplay between both diffusive and advective modes of drug transport and the design of MF devices, taking into account the geometry of the MF device and porosity of MCTSs. The advective component of drug transport plays an essential role in drug delivery *in vivo*, as the molecular transport of drugs with a molecular mass M_r_ > 1,000 is advection-dominant, as opposed to the transport of low molecular mass drugs with M_r_ < 1,000, which are diffusive-dominant (Dewhirst and Secomb, 2017). To examine this effect, we evaluated the dominant drug transport mechanisms in MF devices with various designs. The CFD model facilitated the assessment of the dominant transport mechanisms of drugs in MF devices with different designs.

Our study shows that it is more favourable to use MF devices with a DSC design that enable more rapid delivery of the model drug (DOX) to the MCTSs, compared to the MF device with a single supplying channel. The leading cause of this effect is attributed to the fact that flow in a two-channel MF device creates a pressure gradient in the microwell that results in a more efficient delivery of drug molecules to the MCTSs than in a single-channel MF device. Given the complexity of finding the relationship between various coupled (interdependent) geometrical parameters and non-geometrical conditions, as well as the significance of parametrizing the model by integrating the data into experimental data, we developed hierarchically clustered heatmaps to provide guidance for interpreting data and designing and designing new experiments for spheroid-on-a-chip platforms.

## Conclusion

We have described a novel computational framework involving governing physical equations of fluid flow and transport, parametric sweeping, parallel computing, and post-processing and analysis that enables simultaneous simulation of >15,000 flow scenarios and designs of a spheroid-on-a-chip MF platform. While the approach is general and can be applied to any MF design, our study focused on one particular spheroid-on-a-chip configuration, which we used as a case study to illustrate the power and efficiency of our approach. The proposed simulation model has the potential to be extended to studies of other types of MF organ-on-a-chip devices. Moreover, the proposed framework will be helpful as a pipeline for more complex CFD modelling and optimization near future.

## Data Availability

The original contributions presented in the study are included in the article/Supplementary Material, further inquiries can be directed to the corresponding author.
