# Supplementary material for: Computational Modelling and Big Data Analysis of Flow and Drug Transport in Microfluidic Systems: A Spheroid-on-a-Chip Study
Source: Front Bioeng Biotechnol. 2021 Nov 23;9:781566. doi: 10.3389/fbioe.2021.781566 (PMC8650096; doi:10.3389/fbioe.2021.781566)
Supplement: Supplementary file 1 [file DataSheet1.docx]

Supplementary Material

Computational Modelling and Big Data Analysis of Flow and Drug Transport in Microfluidic Systems: A Spheroid-on-a-Chip Study

Sina Kheiri^1^, Eugenia Kumacheva^2,3^, Edmond W.K. Young^1,3*^

^1^Department of Mechanical & Industrial Engineering, University of Toronto, Canada

^2^Department of Chemistry, University of Toronto, Canada

^3^Institute of Biomedical Engineering, University of Toronto, Canada, University of Toronto, Canada

*** Correspondence:**Edmond W.K. Young, Ph.D., P.Eng., F. CSME

Associate Professor

Department of Mechanical & Industrial Engineering

Institute of Biomedical Engineering

University of Toronto, Toronto, ON, Canada

E-mail : eyoung@mie.utoronto.ca

Phone: +1 (416) 978-1521

# Supplementary Note 1: Design and Fabrication of Microfluidics (MF) Device

Figure S1 shows the schematic of the spheroid-on-a-chip microfluidic (MF) arrayed device used in our physical experiments. The MF device was fabricated using soft lithography techniques.(Xia and Whitesides, 1998) After mixing polydimethylsiloxane (PDMS) elastomer and curing agent (at a weight ratio of 10:1), the mixture was poured over a silicon wafer mould in a petri dish. After degassing, the mould was placed in an oven at 70 °C for three hours to cure the poured PDMS. The PDMS was peeled from the mould and was bonded to a PDMS-coated glass slide using air plasma treatment for 90 seconds. The final device was placed in a 115℃ oven overnight.

# Supplementary Note 2: Formation, growth and, characterization of MCF-7 breast cancer spheroids.

## Cell culture

MCF-7 cells were cultured in T-75 tissue flasks with 10 mL complete cell culture medium at 37℃ and 5% CO2 in the incubator_._ Complete cell culture medium consisted of DMEM-F12, 10%(v/v) fetal bovine serum, 1% (v/v) penicillin/streptomycin, and 5-mg insulin. At approximately 90% confluency, the cells were passaged using 4 mL trypsin-EDTA solution for 15 min, followed by 5 mL of cell culture medium. The cell pellet was then re-suspended in 1 mL of the cell culture medium, and 300 µL of the cell suspension was transferred into a tissue flask.

## Generation of spheroids in MF device

The MF device was first washed for 20 min with fluorinated oil with 0.1 wt.% triblock copolymer perfluoropolyether and poly(ethylene oxide-co-propyleneoxide) surfactant at a flow rate of 0.1 mL/hr. Next, the fluorinated oil solution was replaced with MCF-7 cells-in-hydrogel (1 wt.% Aldehyde-functionalized CNCs and 2 wt.% gelatin(Prince et al., 2018)) suspension, which was infused for 10 min. Cell density was 1.2×10^5^ cells/µL in the hydrogel. Then, the cell-in-hydrogel suspension was changed with fluorinated oil to form cell-laden droplets in the microwells. Next, the MF device was sealed with PCR sealing tape and incubated at 37 °C for 1.5 hours, allowing the hydrogel to cross-link. The droplets were then cultured with a complete culture medium continuously using syringe pumps at a 0.002 mL/hr flow rate, while the drug was supplied using higher flow rates (0.01 mL/hr and 0.02 mL/hr). **Figure S2** summarizes the steps for the formation and growth of MCF-7 cell spheroids.

## Viability test of MCF-7 spheroids

Viability of spheroids was characterized using a live/dead staining viability kit. NucGreen/NucBlue viability kit (Invitrogen^TM^) was used to stain cell nuclei as blue and dead cells as green. The dye solution was mixed as recommended (2 drops of each dye per 1mL of cell culture medium) and loaded into the microchannel at a flow rate of 0.1 mL/hr. Stained spheroids were imaged using an epifluorescence microscope (Nikon Inverted Microscope Eclipse Ti-S). Quantification of cell viability was carried out using the following equation:

$$Cell viability=\frac{n_{nuclei}-n_{dead}}{n_{nuclei}}$$

## Immunofluorescence staining of spheroids on MF device

Staining was conducted by perfusing a series of solutions at a flow rate of 0.15 mL/hr. into the device. First, the cell culture medium was washed out by infusing 1X HBSS for 20 minutes. Next, 5% formalin (in 1X HBSS) was purged for 1 hour to fix the cells. Then formalin was replaced by 0.1 M glycine in 1X HBSS for 1hour. 0.5% triton-X in 1X HBSS was subsequently infused for 30 minutes to permeate the spheroids. Immunofluorescence (IF) solution (consisting of 0.05 wt.% NaN_3_, 0.1 wt.% Bovine Serum Albumin, 0.2 vol.% Triton-X-100 and 0.05 vol. % Tween-20 in HBSS) was perfused for 45 minutes. Afterwards, the primary blocking solution (10 wt.% goat serum in IF washing solution) was infused for 1 hour. Lastly, primary antibodies (Alexa Fluor 488 E-Cadherin rabbit monoclonal antibody and Alexa Fluor 568 Phalloidin) were diluted 1:800 in the primary blocking solution and perfused for 1 hour. The MF device was incubated overnight at 4℃. Lastly, DAPI (0.5 ng/mL in 1X HBSS) was perfused for 1 hour to stain the cell nuclei. The stained spheroids were imaged using Zeiss LSM700 confocal microscope.

# Supplementary Note 3:Parallel computing and post-processing setup.

Because the CAD Compute Cluster utilizes a “batch job” system, the simulation should be carried out using IBM Spectrum Load Sharing Facility (LSF) Suite for HPC. After preparing the skeleton of the CFD simulation, the file was transferred to CAD Compute Cluster using FileZilla. To run the process of solving the equations, the following script was used to run the simulation in batch mode:

- *#!/bin/sh*
- *# embedded options to bsub - start with #BSUB*
- *# -- Name of the job --*
- *#BSUB -J COMSOL_example*
- *##BSUB -W 04:00*
- *# -- specify the number of processors --*
- *#BSUB -n 8*
- *# -- specify the number of nodes --*
- *##BSUB -R "span[hosts=5]"*
- *# -- user e-mail address --*
- *##BSUB -XXX@mail.XX*
- *# -- mail notification --*
- *# -- at start --*
- *##BSUB -B*
- *# -- at completion --*
- *##BSUB -N*
- *# -- Specify the output and error files. %J is the job ID --*
- *# -- -o and -e mean append, -oo and-eo mean overwrite --*
- *#BSUB -oo TEST_%J.out*
- *#BSUB -eo TEST_%J.err*

## Hierarchically clustered Heatmap in Python

All the CFD model data were exported and saved in .xlsx. The .xlsx file was then imported to Spyder 4.2.1 development environment. The *seaborn* library was used for processing and plotting the results. The hierarchically clustered heatmap of the matrix dataset was carried out using *seaborn.clustermap()* function.

## Spheroid-chip Investigation by Numerical modelling-Application Interface (SINAI)

The developed standalone user-friendly application can be installed and executed on different operating systems, including Windows, macOS, and Linux. Figure S3 shows the SINAI interface. The installation file can be found here:

[**Windows**](https://drive.google.com/drive/folders/1QsETq4bDtDMPK3E-9SkCHb2N33DQjXNs?usp=sharing)

[**macOS**](https://drive.google.com/drive/folders/1-xqTfeCAILo6sGqv7E7xtRnXv7FKYJaM?usp=sharing)

[**Linux**](https://drive.google.com/drive/folders/1let_9SJHwa5dlu3Bxk4ZpEdb3AohMpPW?usp=sharing)

# Supplementary Note 4: Comparison of experimental and CFD Dox uptake

We used image processing algorithms to measure the drug concentrations/intensity to benchmark the drug uptake from CFD simulations and experimental results. For experimental results, the measurement was carried out using the mean intensity of fluorescence images, focused on the spheroid mid-plane, for different spheroids under two different flow rates (0.01 and 0.02 mL/hr), as shown in **Figure S6A**. For CFD simulation results, a cut-plane was defined at the center of the porous medium (**Figure S6B**), and the total flux of the drug was evaluated as shown in **Figure S6C**. Next, the results of CFD simulations were measured using image processing algorithms. The experimental results suggested that the drug uptake under a higher flow rate (Q=0.02 mL/hr) is ~2.7 times higher compared to a low flow rate (Q=0.01 mL/hr). The results from CFD modelling suggested that the drug uptake is ~2.3 higher when the flow rate is set to be Q=0.02 mL/hr. No significant difference was observed after the t-test for the experimental results with the CFD simulation (*p*>0.05). The developed CFD model is missing several factors (e.g., biological interactions); however, the results suggested that the modelling can simulate the drug uptake with relatively high accuracy.

## Supplementary Figures

**
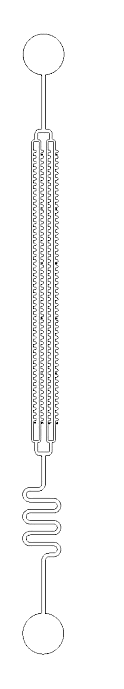
**

**Fig. S1. Design of the MF device.** Schematic of the MF device that includes an array of 200 microwells. Each row contains 50 microwells of 100-µm diameter. The inlet and outlet are labelled as 1 and 2, respectively.

**
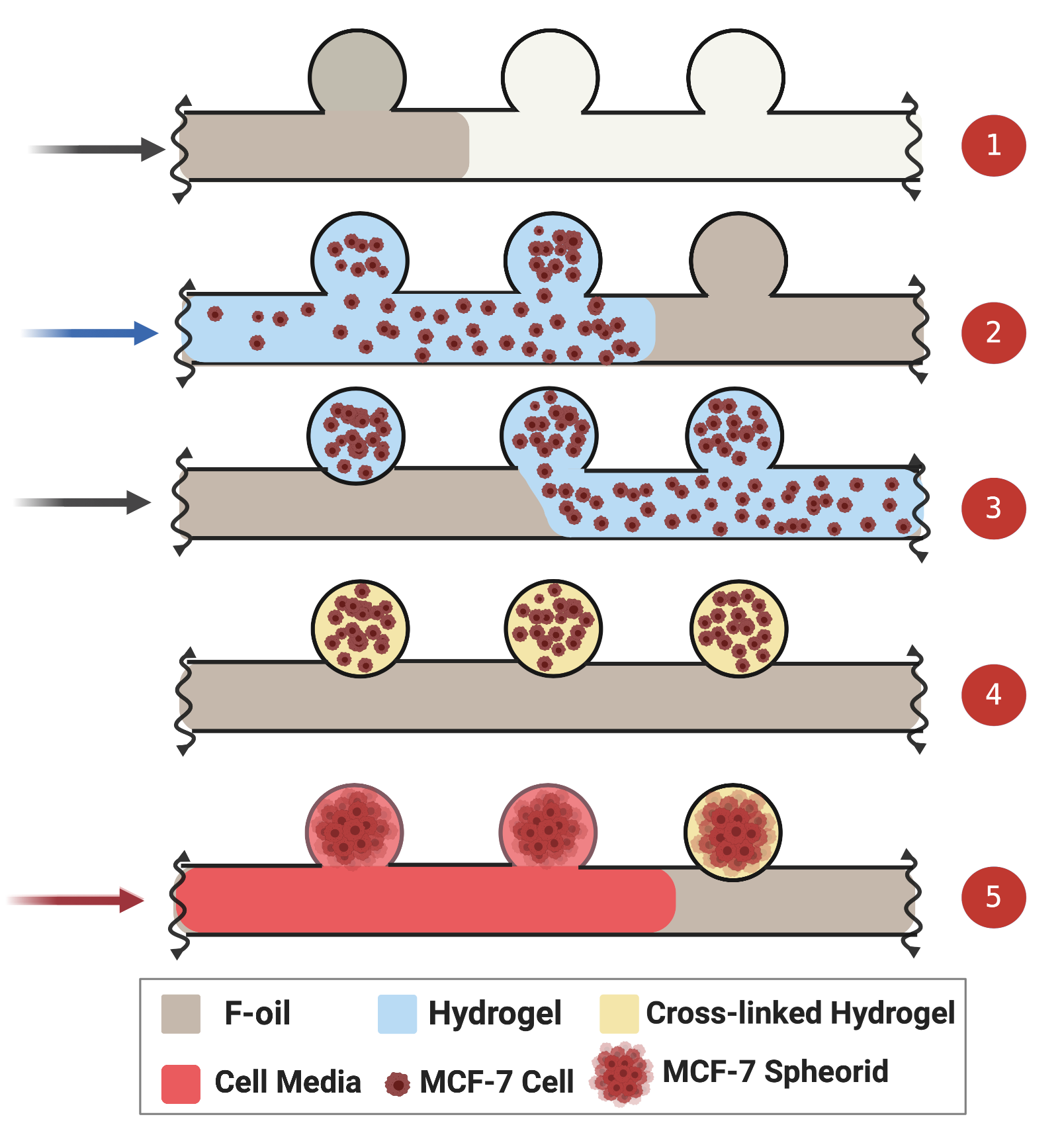
**

**Fig**. **S2.** Schematic of required steps for formation of spheroids on MF device. The arrows show the infusion of the liquid (fluorinated oil, cell media, or hydrogel precursor). (1) Washing of the MF device with fluorinated oil. (2) Loading of the cell-hydrogel precursor. (3) Replacement of cell-in-hydrogel suspension with fluorinated oil to form cell-laden droplets in the microwells. (4) Incubation of the MF device to cross-link cell-laden droplets. (5) Perfusion of cell culture medium to allow spheroid growth.


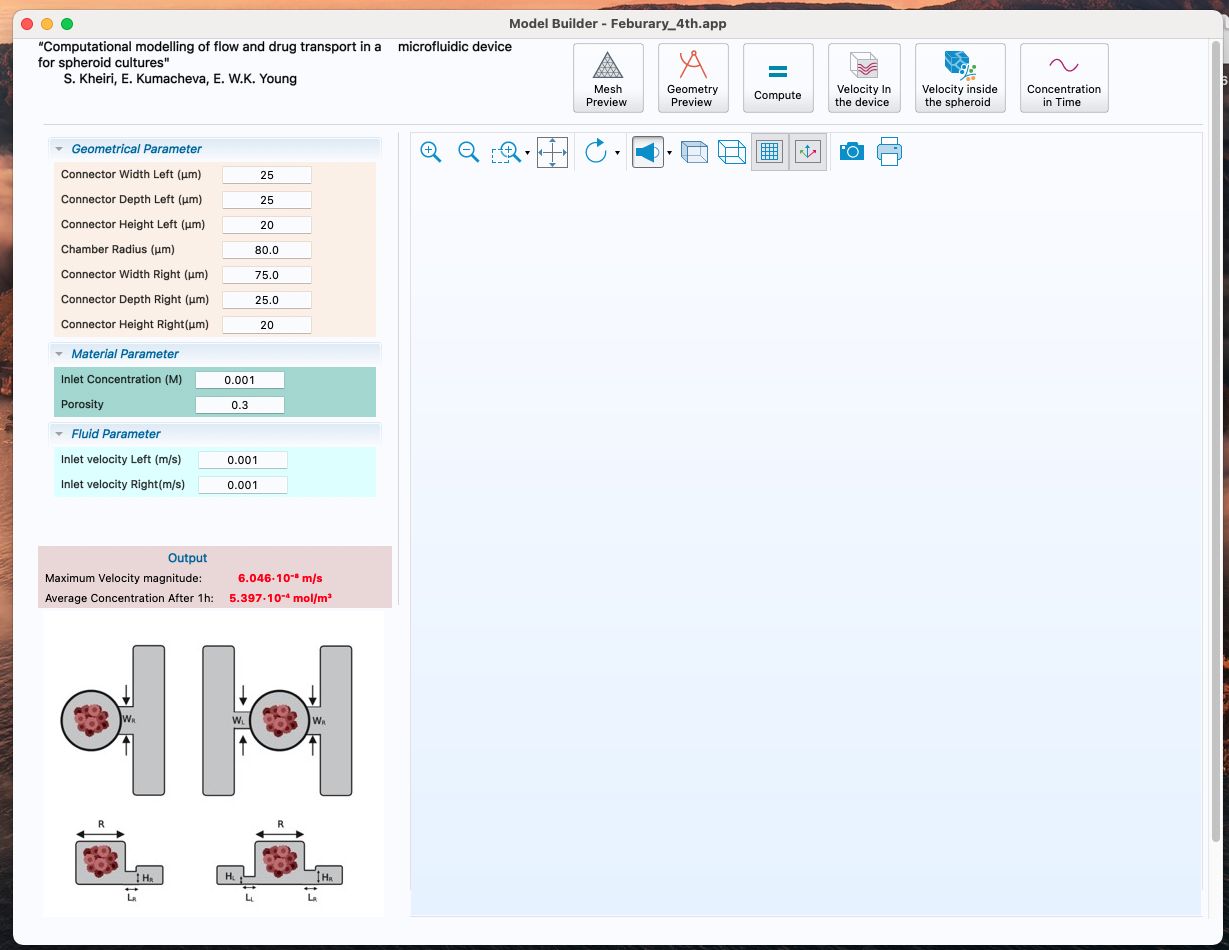

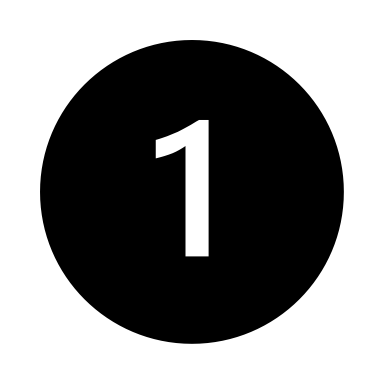

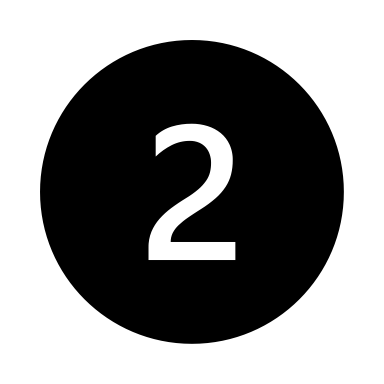

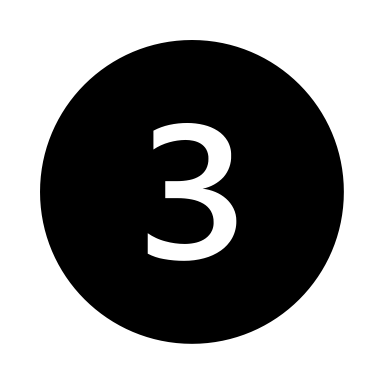

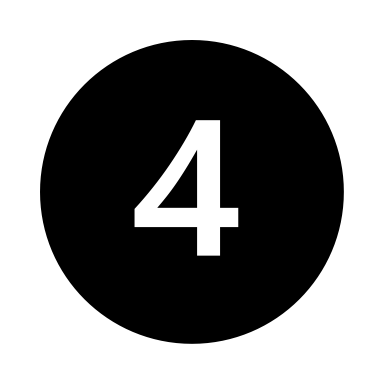

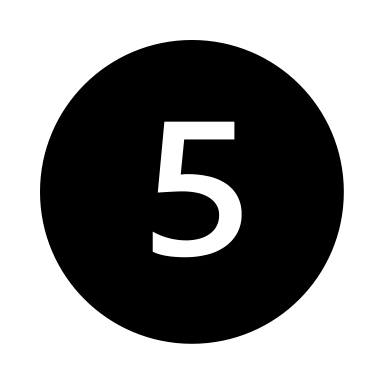


**Fig. S3**. The SINAI user interface. (1) The configuration section. (2) Function buttons. (3) View adjustment toolbar. (4) Final output data. (5) The instruction graph for choosing the parameters.


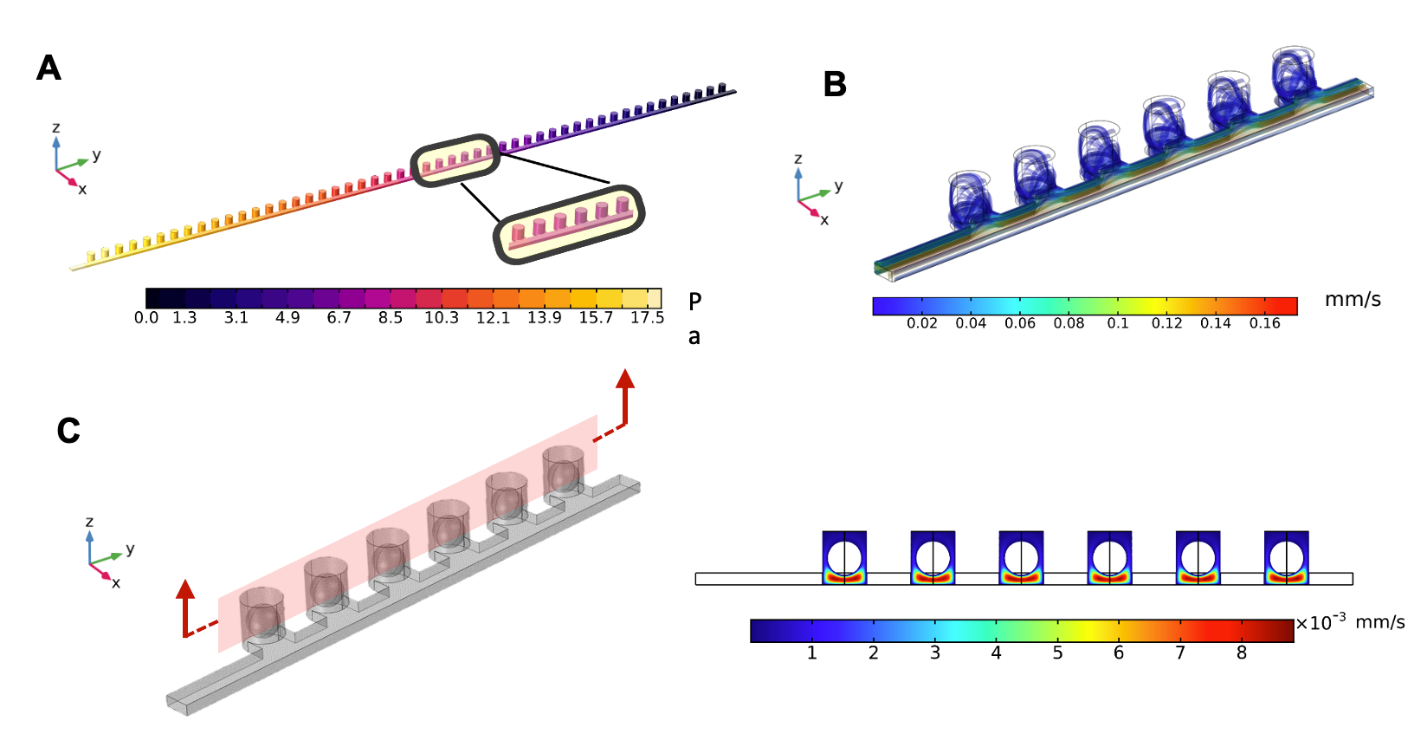


**Figure S4. Simulation results on the complete microfluidic device.** (A) Pressure gradient volume contours in a single row of microwells. (B) Fluid flow velocity streamlines in the microchannel and six microwells corresponding to the box shown in (A). (C) The defined cross-sectional contour of velocity magnitude inside the microwells corresponding to the box shown in (A).


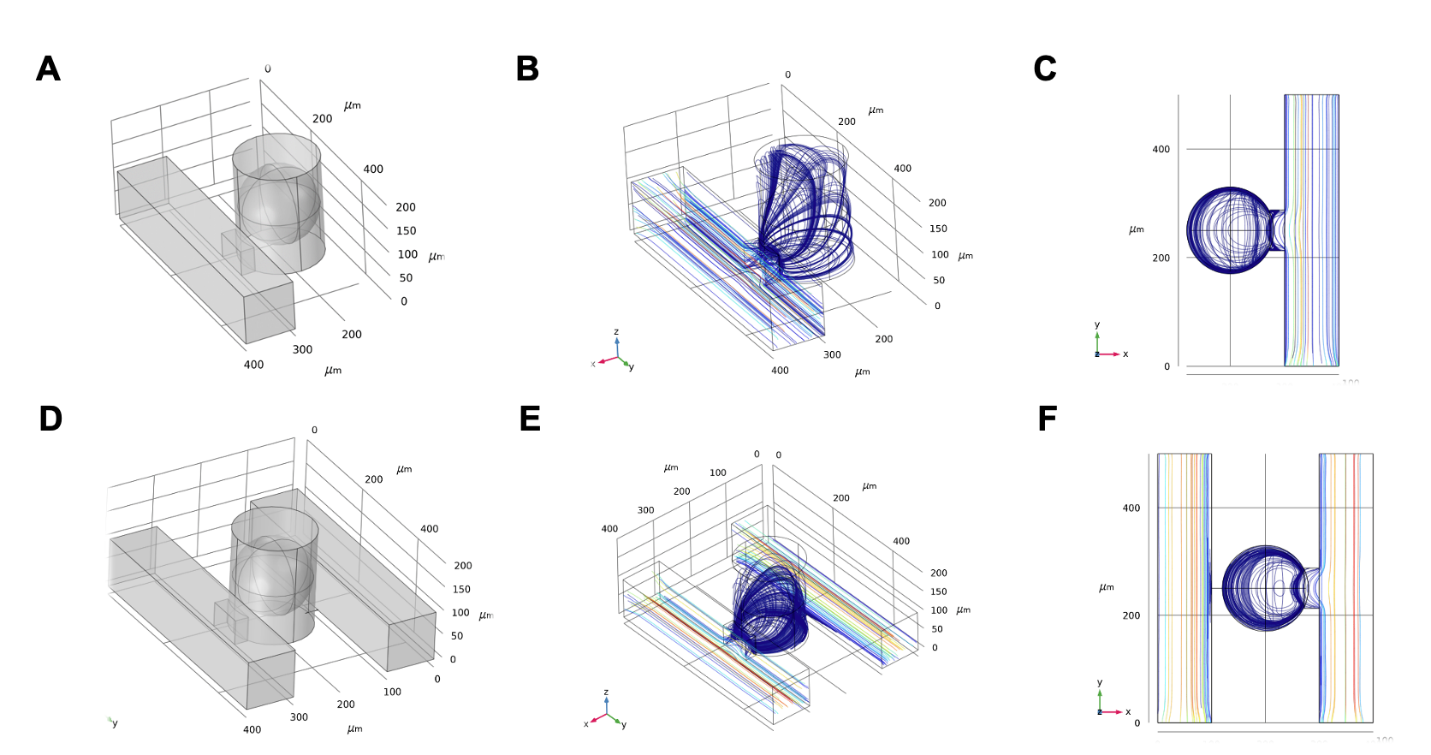


**Figure S5**. Comparison of fluid flow simulation in single-channel model and double-channel. (A) The defined geometry of single supply channel (SSC) design in the benchmark CFD model. (B-C) Velocity streamlines in the microchannel and microwell of SSC design. (E) The defined geometry of double supply channel (DSC) design in the single dataset CFD model. (E-F) Velocity streamlines in the microchannel and microwell of DSC design.

**
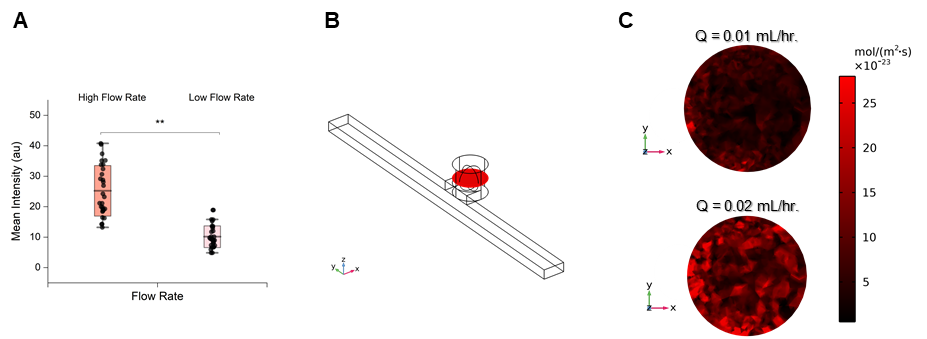
**

**Figure S6**. Comparison of doxorubicin uptake in experimental and CFD simulation results. (A) Mean intensity of doxorubicin fluorescence results in spheroid mid-plane, where Q= 0.01 mL/hr (low flow rate) and 0.02 mL/hr (high flow rate) (*n=30, **p<0.05*). (B) Defined cut-plane in COMSOL for evaluation of doxorubicin flux in mid-plane. (C) Results evaluation of total dox flux on the midplane when Q= 0.01 mL/hr. and 0.02 mL/hr.


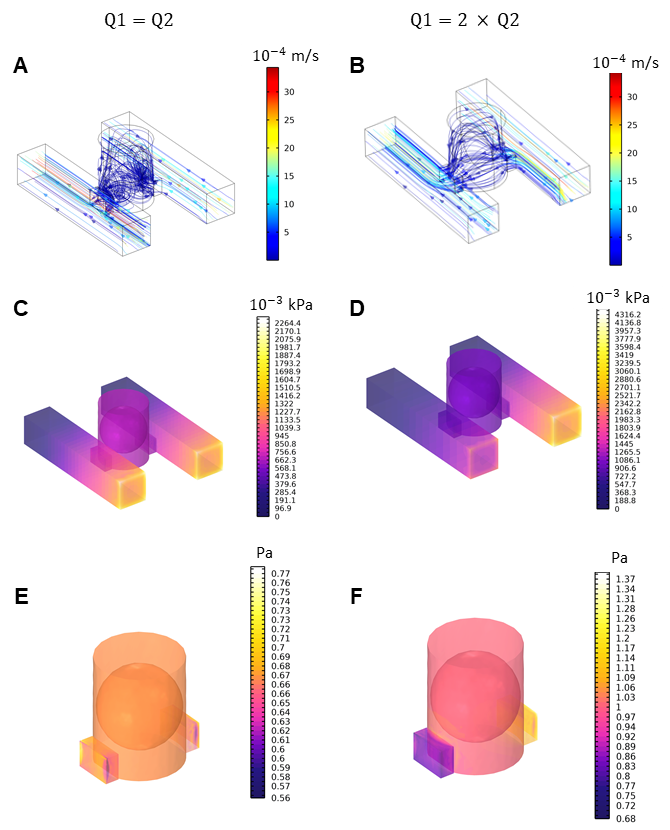


**Figure S7**. Comparison of fluid flow simulation in the double-channel CFD model when Q_1_=Q_2_ and Q_1_= 2 × Q_2_. (A-B) Velocity streamlines in the microchannel and microwell. (C-D) Pressure gradients along the microchannel and microwell. (E-F) The pressure gradient in the microchamber.


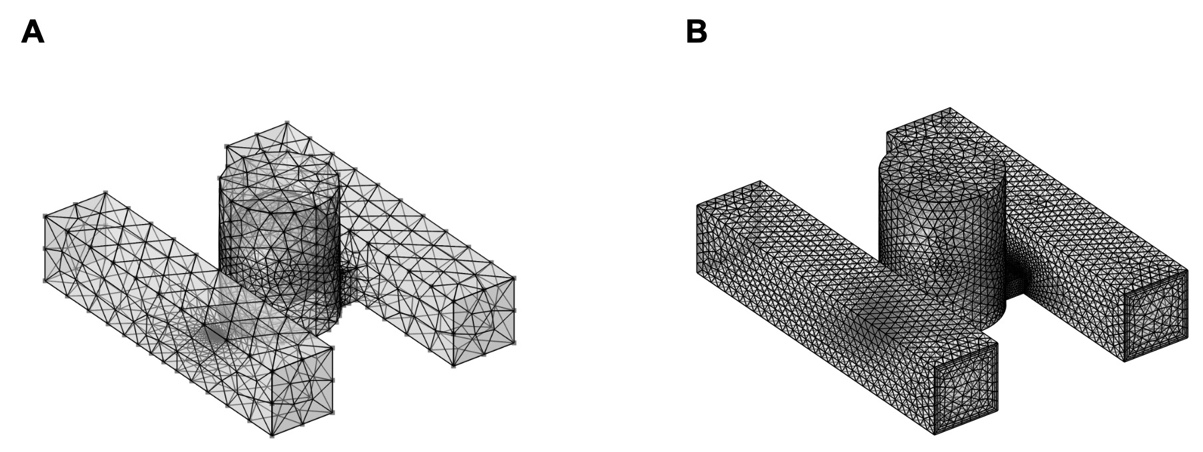


**Figure S8**. Comparison of solution-adaptive mesh refinement (A) the first generated mesh before adaptive refinement, (B) results of four iterations of solution-adaptive mesh.

# Tables

**Table S1.** Summary of parameters for simulation of the parametric sweeping

| **Software and solver** |  |
| --- | --- |
| Geometry software | COMSOL Multiphysics |
| Physics types (COMSOL) | Creeping flow and Transport of Diluted species |
| Study types (Transient or Steady) | Time Dependent |
| Solver | Fully Coupled (Direct) |
|  |  |
| **Mesh** |  |
| Number of elements | 720 000 - 1600 000 |
| Mesh Type | Adaptive and calibrated for fluid dynamics |
|  |  |
| **Flow properties** |  |
| Material | Water |
| Viscosity | 0.001 Pa s |
| Density | 1000 kg/m^3^ |
|  |  |
| **Porous matrix properties** |  |
| Material | Spheroid (manually defined)(Prince et al., 2018) |
| Porosity | 0.2-0.9 |
| Permeability | $8\times{10}^{-11}$cm^2^ |
| Diffusivity method | Millington-Quirk |
|  |  |
| **Drug properties** |  |
| Material | Dox (manually defined) (Zhao et al., 2013) |
| Molecular weight | 543.52 g/mol |
| Diffusion coefficient | $4\times{10}^{-10} m^{2}/s$ |
| Concentration | 10 µM |
|  |  |
| **Boundary conditions** |  |
| Inlet flow condition | Fully developed – averaged velocity flow rate |
| Inlet transport condition | Constant concentration (1 and 0.125 μM) |
| Outlet condition | Constant pressure (0 Pa, gauge) |
| Walls | No slip and no flow-through conditions |

# References

Prince, E., Alizadehgiashi, M., Campbell, M., Khuu, N., Albulescu, A., De France, K., et al. (2018). Patterning of Structurally Anisotropic Composite Hydrogel Sheets. *Biomacromolecules* 19, 1276–1284. doi:10.1021/acs.biomac.8b00100.

Xia, Y., and Whitesides, G. M. (1998). SOFT LITHOGRAPHY. *Annu. Rev. Mater. Sci.* 28, 153–184. doi:10.1146/annurev.matsci.28.1.153.

Zhao, S., Zhao, H., Zhang, X., Li, Y., and Du, Y. (2013). Off-the-shelf microsponge arrays for facile and efficient construction of miniaturized 3D cellular microenvironments for versatile cell-based assays. *Lab Chip* 13, 2350. doi:10.1039/c3lc50183c.
